# Feasibility of Collecting Umbilical Cord Blood in Jordan and the Effect of Maternal and Neonatal Factors on Hematopoietic Stem Cell Content

**DOI:** 10.4084/MJHID.2014.019

**Published:** 2014-02-18

**Authors:** Ayad Ahmed Hussein, Randa M. Bawadi, Lubna H. Tahtamouni, Haydar Frangoul, Ali Z. ElKarmi

**Affiliations:** 1 Bone Marrow and Stem Cell Transplantation Program, King Hussein Cancer Center, Amman, Jordan; 2 Department of Biology and Biotechnology, Faculty of Science, The Hashemite University, Zarqa, Jordan; 3 Pediatric Stem Cell Transplant Program, Monroe Carell Jr. Children’s Hospital at Vanderbilt, Nashville, TN, United States

## Abstract

**Background:**

Cord blood transplant is an accepted treatment for many malignant and non-malignant diseases. We sought to determine the feasibility of collecting cord blood in Jordan and the effect of maternal and fetal factors on the quality of the cord blood units.

**Methods:**

A total of 124 cord blood units were collected, and 75 (60%) cord blood units were included in this analysis. Cord blood volume, total nucleated cell (TNC) count, cell viability and CD34^+^ content were measured, and clonogenic assay was performed.

**Results:**

The mean volume of the collected units was 68.9 ml (range 40–115) with mean nucleated cell count of 6.5 x 10^8^ (range 1–23.0). Our results showed a positive correlation between the volume of cord blood and TNC count (p=0.008), cell viability (p=0.001), CD34^+^ content (p=0.034) and the length of the umbilical cord (p=0.011). In addition, our results showed an inverse relation between the Colony Forming Unit-Granulocyte Macrophage (CFU-GM) concentration and the gestation duration (p=0.038).

**Conclusion:**

We conclude that it is feasible to collect cord blood units in Jordan with excellent TNC and CD34^+^ cell content. The volume of cord blood collected was associated with higher TNC count and CD34^+^ count. Efforts toward establishing public cord blood banks in our area are warranted.

## Introduction

Hematopoietic stem cell transplant (HSCT) is a well-established therapy for various malignant and non-malignant diseases in adult and children. Bone marrow was the main source of hematopoietic stem cells for decades. Recently, more commonly used sources include peripheral blood and umbilical cord blood. Umbilical cord blood (UCB) emerged as a new source for hematopoietic stem cells (HSC) in the early 1988.^1^ A major advantage for UCB as a stem cell source for allogeneic HSCT is its immediate availability. Additionally, the naive nature of its lymphocytes led to decreased risks of graft versus host disease (GVHD) and allowed for successful HLA mismatched transplant with low rates of acute and chronic GVHD.^2^–^4^ This has resulted in UCB being a widely used source for HSCT to treat many malignant and non-malignant diseases.

Higher numbers of total nucleated cells (TNC) and CD34^+^ cells in the UCB units have resulted in faster and more sustained engraftment and improved survival following cord blood transplant.^3^,^4^ Several studies from Europe,^5^–^7^ Japan,^8^ Taiwan,^9^ and the United States^10^,^11^ have examined the various factors that can improve the quality of the collected UCB units. Some of the variables that were identified included maternal-related factors such as mother age, race, number of previous births and smoking status, and fetal-related factors such as weight, sex, birth order, placental weight and umbilical cord length. The rational being is that it would be useful to predict UCB cell content using information of donor-related variables before collection and cell processing.^7^

None of the above cited studies were performed in any of the Middle Eastern countries, despite the fact that there are unique demographic and genetic differences in patients in this region.^12^ In the current study we sought to investigate the feasibility of collecting UCB and the effect of different maternal and fetal variables that might have an impact on the hematopoietic parameters of UCB in Jordan. According to our knowledge, this is the first study to be conducted particularly in the Arab region and in Jordan.

## Materials and Methods

### Umbilical cord blood collection

Between August 2010 and July 2011, 177 mothers delivering their babies at Al-Isra’a hospital, Amman, Jordan, were approached to participate in this prospective study. One hander and twenty-four mothers (70%) agreed to participate and signed a consent form. The UCB was collected exclusively from term (gestation period 37–42 weeks) single-birth babies born through normal vaginal delivery. Cord blood was collected after the baby was delivered but before the delivery of the placenta. A regular blood-donor set was used for UCB collection containing 28 ml citrate phosphate dextrose-adenine (CPD-A) anticoagulant. The collection was performed by the obstetrician delivering the baby and not by a trained technician. The umbilical cord (UC) was sterilized with povidone iodine in a unidirectional move, and 16-gauge needle of the prepared blood-donor set was inserted into the umbilical vein. Blood was allowed to flow by gravity, and the needle was removed when blood flow ceased as has been previously described.^6^,^10^ The study design and UCB collection procedure was approved by the Hashemite University, and Al-Isra’a general hospital Institute Review Boards.

### Evaluation of umbilical cord blood parameters

For the current study, UCB units were deemed unacceptable if the total volume collected was less than 30 ml and/or if the unit was delivered for analysis past 24 hours of collection. The UCB units were processed and analyzed in the biology laboratory at the Hashemite University, Jordan. The UCB was incubated with FITC-conjugated anti-CD45 fluorescein (MACS, Germany) and PE-conjugated anti-CD34 PE (MACS, Germany) for 30 min at room temperature in the dark. After incubation, RBCs were lysed with the lysis solution (Coulter, France) and then washed twice with 10% bovine serum albumin (BSA) in phosphate buffer saline (PBS). For each tube, 20,000 live events were counted in a flowcytometer counter (Partec, Germany). CD34^+^ cells were selected based on their forward- and 90°-scatter properties and dim CD45 expression.^13^ The clonogenic assay (CFU-GM assay) was performed as described previously.^14^ Briefly, mononucleated cells (MNCs) were cultured at 1.0 X 10^5^/ml in RBMI-1640 medium (MACS, Germany) containing 0.8% methylcellulose (Sigma Aldrich, USA), 20% fetal bovine serum (FBS; Lonza, Belgium), 450 μg/ml human transferrin, 10 ng/ml GM-CSF, 10 ng/ml IL-3 (Stem Cell Technologies), and 1% BSA. Cells were incubated at 37°C in a humidified atmosphere of 5% CO_2_ for 14 days. Colonies (clusters containing at least 50 cells) were counted using an inverted microscope (Leica, Germany). Viability was determined using trypan blue dye exclusion method, where the non-viable cells stain deep blue.

### Maternal and neonatal data collection

Data regarding maternal age, the number of previous pregnancies and live births were collected from the medical files. Neonatal data such as the weight of the baby and the placenta, baby’s gender, and UC length were collected from the obstetric staff clinical notes at Al-Isra’a general hospital. A standard questionnaire was prepared and used for data collection.

### Statistical analysis

Statistical analysis was carried out using STATISTICA 7 analysis program (StatSoft Inc., OK, USA). Results were expressed as mean ± standard deviation (SD). One-way analysis of variance (ANOVA) was used to test for a significant difference between mean values of all. Spearman’s correlation was used to assess the association between the different variables. A p value of ≤ 0.05 was considered statistically significant.

## Results

### Characteristics of the study population

A total of 177 prospective mothers were approached to participate in the current study, 53 (30%) of them refused to participate due to cultural and/or lack of knowledge regarding benefits of UCB and safety of the collection procedure. 124 units were prospectively collected for this study. In 17 (13.7%) UCB units the net volume of cord blood was less than 30 ml, in 23 (18.5%) units some maternal and/or neonatal data were missing, and in 9 (7.3%) units the samples were not delivered for the laboratory within 24 hour of collection. A total of 75 UCB units (60.5% of the total collected units) were included and analyzed in this study. The characteristics of the donating mothers and babies are shown in Table 1. The mean maternal age was 28 years (range 19–43). Thirty three percent of the donating mothers were delivering their first babies, 21.3% second, 20% third and 25.3% fourth or more. Fifty one percent of the delivered babies were males, and 49% were females. The mean weight of the delivered babies was 3178 gm (range 220–4160), and mean placenta weight was 526 gm (range 400–655). Seven percent of the donating mothers reported that they were current smokers.

### Analysis of umbilical cord blood samples

Cord blood cell counts were analyzed within 24 hours of collection. The mean volume of UCB collected (not including the 28 ml of anticoagulant) was 68.9 ml (range 40–115 ml). The mean viability was 94.9% (range 80–99%), the mean total nucleated cell (TNC) count was 6.5 x 10^8^ (range 1–32**),** with 10.6% have TNC of more than 1 x 10^9^ and 4% of more than 1.2 x 10.^9^ The mean total mononuclear cell count (MNC) was 3.4 x 10^8^ (range 0.5–14.9), the mean total CD34^+^ cell count was 3.8 x 10^6^ (range 0.2–11.8), and the mean total CFU-GM was 9.9 x10^5^ (range 2–25). The UCB unit’s data are summarized in Table 2.

The results of the univariate analysis correlation are presented in Table 3. The volume of UCB units collected was positively correlated with TNC (p=0.008), cell viability (p=0.001), MNC (p=0.018), CD34^+^ cell count (p=0.034) and with the umbilical cord length (p=0.011). There was also a trend towards obtaining higher UCB volume from mothers with increasing number of prior live births (p=0.086). Our results showed that higher TNC is correlated with MNC (p=0.001), CD34^+^ cell count (p=0.009), and increased viability (p=0.001). Finally, our study demonstrated an inverse correlation between CFU-GM concentration and the gestation duration (P = 0.038). There was no significant effect of gestational age on TNC or CD34^+^ cell count of the collected UCB.

## Discussion

This is the first study to show that collection of cord blood is feasible and can result in adequate TNC collection and viability in a developing country in the Middle East. Of the 124 women enrolled in the study, the umbilical cord blood of only 17 (13.7%) did not contain adequate volume of blood despite the fact that untrained technicians were present at the delivery to collect the UCB. An additional 25.8% of the UCB units had to be excluded either because some data were missing, or because the UCB did not reach the lab in the required time for processing. Among the 75 units that met the predefined eligibility criteria, the volume, nucleated cell dose and CD34 count was similar to what has been previously published.^5^,^7^,^8^,^10^,^14^

Previous studies concluded that UCB yield of TNC, CD34^+^ cells, and CFU-GM is influenced not only by neonatal and maternal factors but also by ethnicity of the parents.^7^,^15^ In this study, a total of 75 cord blood samples from Jordanian neonates were analyzed in order to investigate any neonatal and maternal factors that might influence UCB unit in terms of TNC and CD34^+^ cell content, and CFU-GM yields. In the current study, the average age of the donor mothers included was 28 years and both the TNC and CD34^+^ cell yield was not influenced by maternal age. While the majority of published data showed similar observation, the study by Nakagawa *et al.* which analyzed 572 UCB units, showed that increasing mother’s age was associated with lower TNC cell yield.^5^,^8^–^10^ We found no significant correlation between birth order and UCB volume, TNC, and CD34^+^ levels. Our findings are different from prior studies which showed that women with few previous live births produced UCB units with higher TNC yields.^9^,^10^

The volume of UCB collected was not influenced by any of the maternal or neonatal factors except the umbilical cord length, as longer cords were associated with higher volume collected. This correlation has only been observed in one prior study from Japan.^8^

We also found an inverse correlation between the CFU-GM concentration and gestational age, which indicates that there is a loss of hematopoietic potential with a longer gestational age. In a study by Ballen *et al.* of 1269 UCB units, there was an 11% decrease in CFU-GM with each additional week of gestation.^10^ Since TNC and CD34^+^ cell counts are the most important predictors of the outcome following cord blood transplant, collecting units with a larger volume is desired. In our current study, the only positive predictor of improved cell count is the volume of UCB collected.

The chance of finding a matched related donor in Jordan is significantly higher than what has been observed in other countries (65% versus 25%) due to more homogeneous ethnic group in the region.^16^

Approximately 10–16% of UBC units collected in the international cord blood banks have TNC of more than 1.2 x 10^9^. In Our study, 10.6% of UCB units collected have TNC of more than 1 x 10^9^ and 4% of more than 1.2 x 10^9^. Although our cell dose was slightly lower than what has been reported by established cord blood banks, this is dependent on the experience of the collection staff which always improves with time.

We believe that establishing a cord blood bank in Jordan will further increase the possibility of identifying donors for patients who lack related donor options. Taking into consideration the geographical and cultural similarities between Jordan and its neighboring Arabic countries, a cord blood bank in Jordan will help patients throughout the region. Additional training and better logistical support are needed to collect UCB units in order to decrease the percentage of unacceptable units collected. We need also more efforts towards education of the parents about the benefits and safety of UCB collection, as only 70% of the approached mothers agreed to participate in this study. A proper cost-effective analysis should be carried out before establishing national cord blood banks in countries with limited resources.

In conclusion, we have found that collection of cord blood units in Jordan is feasible and can result in similar cell content compared to other developed countries. Efforts toward establishing public cord blood banks in our area are warranted.

## Figures and Tables

**Table 1:** Maternal and neonatal characteristics

Variable	

Mean mother’s age, year (range)	28 (19–43)

Smoker	
No	70 (93%)
Yes	5 (7%)

Previous live births	
0	25 (33.3%)
1	16 (21.3%)
2	15 (20%)
3	9 (12%)
4+	10 (13.3%)

Mean gestation period, week (range)	38.7 (37–40)

Mean weight of the baby, gram (range)	3178 (2200 – 4160)

Sex of baby	
Male	38 (50.7%)
Female	37 (49.3%)

Mean placenta weight, gram (range)	526 (400–655)

Umbilical cord length, cm (range)	58.8 (35–90)

**Table 2:** Analysis of umbilical cord blood units (No 75)

Variable	Mean ± SD (Range)/CBU	Mean ± SD (range)/TNC
CB Volume (ml)	68.9 ± 11.5 (40 – 115)	-
Viability (%)	94.9 ± 0.4 (80–99%)	-
TNC x 10^8^	6.5 ± 1.9 (1.0–23.0)	*
MNC x 10^8^	3.4 ± 1.1 (0.5–14.9)	0.05 ± 0.01 (0.01–0.17)
CD34^+^ x 10^6^	3.8 ± 1.9 (0.2–11.8)	0.06 ± 0.03 (0.02–0.18)

* TNC/ml = 0.1 ± 0.02, range 0.25 (0.02–0.27)

**Table 3:** Univariate analysis for correlation.

	Volume (ml)		TNC x 10^8^		CD34+ x 10^6^		CFU-GM x10^5^	

Variable	r *	p †	r	p	r	p	r	p

Donor Age	−0.0701	0.550	0.1426	0.222	0.0216	0.854	0.1439	0.218

Smoker								
No	−0.0981	0.402	−0.0134	0.909	−0.0338	0.773	0.0128	0.913
Yes								

Previous live births								
0								
1	−0.0198	0.086	−0.1172	0.317	0.0785	0.503	−0.1194	0.308
2								
3								
4+								

Gestation period	−0.092	0.433	0.0570	0.627	0.0859	0.463	−**0.2401**	**0.038**

Weight of baby	0.1286	0.272	0.0268	0.819	0.0534	0.639	0.0665	0.571

Placenta weight	0.1158	0.323	0.0164	0.889	−0.0361	0.758	0.0471	0.688

UC length	**0.2909**	**0.011**	0.1541	0.187	0.1716	0.141	−0.157	0.178

Sex of baby								
Male	0.1452	0.214	0.0631	0.591	−0.1032	0.378	−0.1678	0.150
Female								

UCB Volume	-	-	**0.3042**	**0.008**	**0.2452**	**0.034**	0.1978	0.089

Viability (%)	**0.3974**	**0.001**	**0.487**	**0.001**	0.0963	0.411	−0.1331	0.255

TNC x 10^8^	**0.3042**	**0.008**	-	-	**0.3005**	**0.009**	−0.1038	0.376

MNC x 10^8^	**0.2715**	**0.018**	**0.9275**	**0.001**	**0.2774**	**0.016**	−0.0483	0.681

CD34^+^ x 10^6^	**0.2452**	**0.034**	**0.3005**	**0.009**	-	-	0.0689	0.557

CFU-GM x 10^5^	0.1978	0.089	−0.1038	0.376	0.0689	0.557	-	-

* r= Spearman Correlation;

† p= p-value
